# Foot-and-mouth disease virus carrier status in Bos grunniens yaks

**DOI:** 10.1186/1743-422X-10-81

**Published:** 2013-03-11

**Authors:** Huiyun Chang, Yanbin Ma, Tong Lin, Guozheng Cong, Junzheng Du, Jinling Ma

**Affiliations:** 1 State Key Laboratory of Veterinary Etiological Biology, National Foot and Mouth Disease Reference Laboratory, Lanzhou Veterinary Research Institute, Chinese Academy of Agricultural Sciences, Lanzhou 730046, China

**Keywords:** FMDV, Yak, Gene mutation, Carrier status

## Abstract

**Background:**

The carrier status of foot-and-mouth disease virus (FMDV) is complicated, and the role of carrier animals in virus transmission is controversial. To investigate the carrier status of FMDV in animals that live in high altitude, Bos grunniens yaks were infected experimentally with FMDV O/Akesu/58.

**Results:**

All of the yaks showed clinical signs of foot-and-mouth disease (FMD). Total antibody levels against FMDV measured by liquid-phase blocking enzyme-linked immunosorbent assay (LPB-ELISA) and antibody levels against nonstructural proteins (NSP) showed dynamic changes. Three of the five yaks were indentified as carrier animals by RT-PCR method, and the OP fluids from carrier yaks can cause cytopathic effect (CPE) on BHK-21 cells. At last, five persistent infection strains were isolated. Nucleotide mutations of *VP1* gene were analyzed.

**Conclusions:**

After infected with FMDV, all of the yaks showed typical clinical signs. Yaks can keep carrier status for at least 8 months. Total antibody levels against FMDV measured by LPB-ELISA and antibody levels against NSP were at high level for carrier yaks. Sequence alignment of the five isolated strains showed obvious gene and protein mutations.

## Background

Foot-and-mouth disease (FMD) is one of the most contagious viral diseases of cloven-hoofed livestock including cattle, swine, sheep, and goat, as well as over 70 species of wild animals. The disease leads to high mortality in young animals, high morbidity, and loss in productivity [1-3]. *Foot-and-mouth disease virus* (FMDV), the first demonstrated filterable agent causing animal disease, is belong to the *Aphtovirus* genus of the *Picornaviridae* family, and contains a single-stranded positive-sense RNA genome of about 8500 nucleotides [4]. As is the lack of error correction mechanism during RNA replication, FMDV has a very high mutation rate ranging from 10^-3^ to 10^-5^ per nucleotide site per genome replication, which results in the presence of seven serotypes, multiple subtypes, and variants [5,6].

Following acute phase of FMDV infection or vaccination, animals may experience persistent infection without clinical signs, and these animals are defined as carrier animals. The duration of the carrier status was influenced by many factors including host species, and breed [7]. Carrier animals are those from which live virus can be recovered longer than 28 days after exposure [8]. Till now, virus isolation from esophageal-pharyngeal (OP) fluids was thought to be the most sensitive method to detect carrier animals [9,10]. Molecular techniques for the diagnosis of persistent infection have been used which offered a potential ability to improve the detection of a low genome copy number in clinical samples. Several assays were described which utilized reverse transcription-polymerase chain reaction (RT-PCR) for the detection of FMDV [11,12]. With PCR and dotblot hybridization, FMDV RNA was detected in probang samples in which virus could be no longer isolated [13-18]. African cattle was shown to excrete FMDV over three years, sheep for up to one year, goats for up to four months, and African buffalo for at least five years [7,19,20]. No FMDV was detected in pigs from the OP fluids which was sampled 28 days after infection, and considered to be non-carriers of FMDV. However, a study of the replication analysis of FMDV in swine lymphoid tissue might indicate a putative carrier stage in pigs [21]. Animals used in previous studies were almost living in a comparative friendly environment in low altitude. The friendly environment was shown as: small one day temperature difference, high oxygen concentration, and proper atmospheric pressure. Phenotype was influenced by gene and environment, so animals in high altitude regions showed different abilities to oxygen concentration and temperature change. Other abilities also showed different, such as the immune response [22,23]. This may result in different evolution pressure on pathogens invading animals.

To investigate whether animals in high altitude regions experience a different carrier status and gene mutation of FMDV, yaks, a special species living in Qinghai-Tibet Plateau were infected experimentally with the O/Akesu/58. OP fluids and blood were sampled for detection of virus and antibodies level. Results showed that yaks can carry FMDV for at least eight months, and nucleotide, amino acid (AA) sequence of VP1 shared some common mutations in isolated strains [11]. What’s more, carrier yaks always had a high antibody level.

## Results

### Clinical signs of yaks

All of the five yaks experienced acute phase, and showed typical FMDV clinical signs. Fever and primary vesicles were detected 18 h after inoculation. Twenty-four hours after inoculation, body temperature was 41.2°C, and the yaks showed more vesicles on the surface of tongue. Forty hours after inoculation, some vesicles began to break up, and vesicular lesions were detected on hoof crown. What’s more, body temperature began to decline. One week after inoculation, yaks came to recovery period.

### Antibody levels during the carrier status and the detection of virus

**Figure 1:**
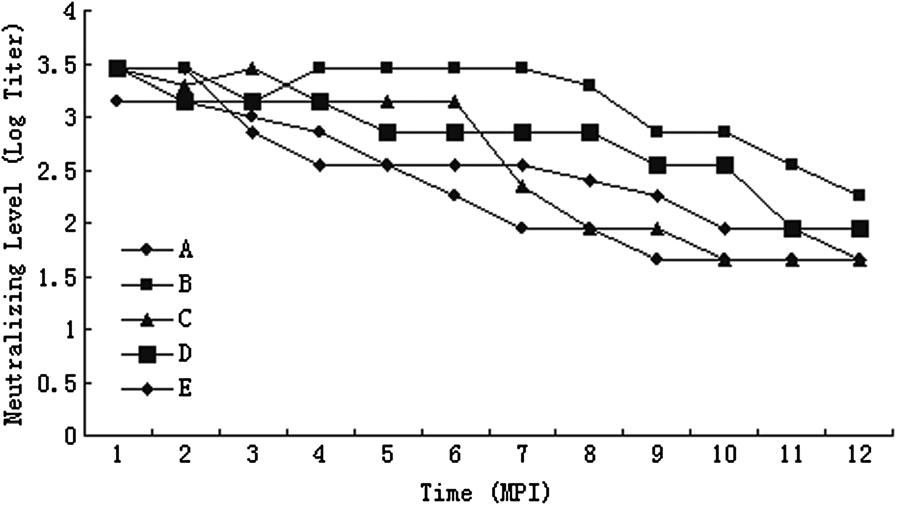
**Total antibodies levels curve of the yaks.** The antibody level was measured by log2 titer of LBP-ELSA. MPI: month post-infection.

**Figure 2:**
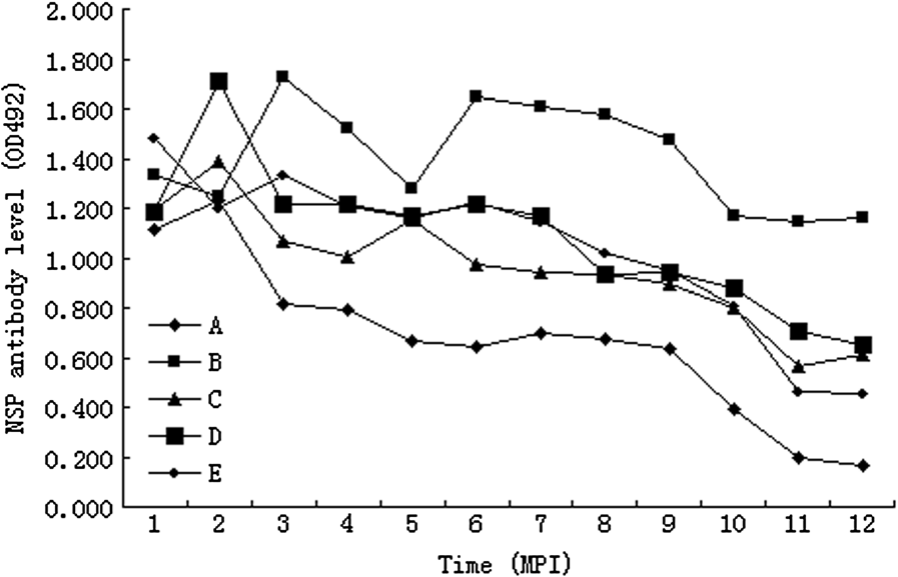
**The 3ABC antibody levels curve of the yaks.** The 3ABC antibody level was measured by the OD value of Indirect-ELISA. A-E indicated the identity of the yaks. And the OD_492_ value of blanking control was 0.2. MPI: month post-infection.

Total antibody levels against FMDV by LPB-ELISA were shown in Figure 1. Total antibodies of the five yaks showed differences between individuals. All of the yaks maintained a high antibody level for at least for 6 months. Total antibody level of yak C had declined obviously since 7 month post-infection (MPI), while yak B maintained a relatively high level until 8 MPI. 3ABC antibody levels of the five yaks were measured by indirect ELISA (Figure 2), and also showed individual difference. Two MPI, 3ABC antibody level reached the peak, and then declined quickly. Yak B kept a high level, while yak A and C showed relatively low antibody levels.

Presence of virus was identified by RT-PCR and CPE (Table 1). The OP fluids were used for RT-PCR detection, and inoculated into BHK-21 cells to isolate the virus strain. From Table 1, we can see that OP fluids which were detected positive by RT-PCR can all lead to CPE within ten times’ cell infection cycle, which indicated the existence of live viruses. No live virus was detected in yak A and C, while yak B kept the longest carrier status. No virus was detected in samples from 10, 11, and 12 MPI. This indicated that carrier period of yaks is at least 8 months.

**Table 1:** The CPE and RT-PCR results of the OP fluids

**MPI**	**A**		**B**		**C**		**D**		**E**	
	**CPE**	**RT-PCR**	**CPE**	**RT-PCR**	**CPE**	**RT-PCR**	**CPE**	**RT-PCR**	**CPE**	**RT-PCR**
1	−	−	**+**	**+**	−	−	−	−	−	−
2	−	−	**+**	**+**	−	−	−	−	**+**	**+**
3	−	−	−	−	−	−	−	−	−	−
4	−	−	−	−	−	−	−	−	−	−
5	−	−	−	−	−	−	−	−	−	−
6	−	−	−	−	−	−	**+**	**+**	−	−
7	−	−	−	−	−	−	−	−	−	−
8	−	−	**+**	**+**	−	−	−	−	−	−
9	−	−	−	−	−	−	−	−	−	−
10	−	−	−	−	−	−	−	−	−	−
11	−	−	−	−	−	−	−	−	−	−
12	−	−	−	−	−	−	−	−	−	−

“**+**” the existence of CPE or the gene was detected by RT-PCR; “−” CPE was not observed or the gene was not detected by RT-PCR. MPI: month post-infection The A-E indicated the identity of the yaks, and 1–12 means the MPI of sampling.

Obviously, there was no certain correlation between antibody level and the existence of virus. But total antibodies and antibody against NSP were at high level for carrier yaks. In this study, when the OD_492_ value of NSP antibody level was above 1.2, and the titer of total antibodies level was higher than 1:708, yak may experience carrier status.

### Nucleotide and AA mutation in VP1

The RT-PCR products of *VP1* gene were sequenced. Pairwise genetic distances of gene and protein sequence distance were shown (Table 2; Table 3). Compared with O/Akesu/58, the isolated strains showed 36 nucleotide mutations. The five isolated strains shared 26 of 36 nucleotide mutations. Fourteen AA mutations were found at translated amino acid sequences. And the five isolated strains shared 8 of 14 AA mutations.

**Table 2:** The gene similarity of aligned sequences

	**Akesu/58**	**B01**	**B02**	**B08**	**D06**	**E02**
Akesu/58	***	95.1	95.1	95.3	95.3	95.8
B01	5	***	99.1	99.2	99.2	99.4
B02	5	0.9	***	99.5	99.5	99.4
B08	4.9	0.8	0.5	***	99.7	99.5
D06	4.9	0.8	0.5	0.3	***	99.5
E02	4.4	0.6	0.6	0.5	0.5	***

The sequences were aligned by Clustal W Method in MegAlign. The upper triangle shows the Percent Similarity of gene sequences of the five isolated strains and O/Akesu/58, while the lower triangle shows the Percent Divergence.

**Table 3:** The protein similarity of aligned sequences

	**Akesu//58**	**B01**	**B02**	**B08**	**D06**	**E02**
Akesu//58	***	94.4	94.8	95.3	94.8	95.8
B01	5.9	***	98.6	99.1	98.6	98.6
B02	5.4	1.4	***	99.5	99.1	99.1
B08	4.9	0.9	0.5	***	99.5	99.5
D06	5.4	1.4	0.9	0.5	***	99.1
E02	4.4	1.4	0.9	0.5	0.9	***

The sequences were aligned by Clustal W Method in MegAlign. The upper triangle shows the Percent Similarity of protein sequences of the five isolated strains and O/Akesu/58, while the lower triangle shows the Percent Divergence.

## Discussion

Similar with previous studies [24,25], yaks can experience acute phase and persistent infection after high-dose FMDV was inoculated. Acute phase or vaccination could cause persistent infection, so some people think that immune status of animals may control virus replication level [1,10,26-28]. Here, we find that yaks detected with the existence of virus always had a high antibody level, which indicated that the existence of high antibody level can protect animals from clinical signs, but not enough to clear virus completely. What’s more, virus was not regularly detected during persistent infection. Obvious gene and protein mutations of VP1 were observed, so we postulated that the variation of VP1 may be a possible reason for carrier status.

The role of carrier animals in virus transmission is undetermined. The transmission from African buffalo to cattle has obtained experimentally which indicated that carrier state can be a source of infection [29,30]. However, no experimental evidence showed that carrier cattle or sheep can transmit virus to uninfected animals. The persistent infection can lead to gene mutation [31], possibly being responsible for the generation of new viral variants in the field. The research of persistent infection and gene mutation of FMDV is a complicated and time-consuming work. From this research, we can conclude that yaks can keep carrier status for at least 8 months. To further study the phenomenon, virulence of the isolated strains to other species and reconstruct of these mutation sits will be the aim of future work.

## Conclusions

After infected with FMDV, all of the yaks showed typical clinical signs. Yaks can keep carrier status for at least 8 months. Total antibody levels against FMDV measured by LPB-ELISA and antibody levels against NSP were at high level for carrier yaks. Sequence alignment of the five isolated strains showed obvious gene and protein mutations.

## Materials and methods

### Virus and virus inoculation

In this study, O/Akesu/58 (serotype O, isolated in Akesu, XinJiang, 1958) which was adapted to suckling mouse and BHK-21 was used. Per Yak was inoculated under the mucosa of tongue with a dose of 10^4^ID_50_ tested in cattle.

### Yaks and treatment

Five 1-year-old yaks (*Bos grunniens*) were used in this study. The yaks were free of antibodies against FMDV, and the OP fluids of yaks were free of FMDV by RT-PCR. The yaks were random named from A to E. The five yaks were all inoculated with O/Akesu/58. The yaks were housed in Biological safety protection third-level (BSL-3) animal homes of Lanzhou Veterinary Research Institute. All tests were approved by the Animal Ethics Committee of the Animal Sciences Group of Gansu Province.

### Tissue samples and treatment

The yaks were sampled at 1 MPI, and kept being sampled monthly for 12 months. Each sample was given an identity number consisting of one letter and two numbers. The letter means the identity of the yak, and the two numbers represents the time of sampling. For example, E03 means that the sample was collected from yak E 3 MPI. Animals were kept being fasted for 12 hours before sampled. Five milliliter OP fluids and 10 ml blood were taken from each yak. The OP fluids were moved into a centrifuge tube with equal volume of PBS/TTE. Then the OP fluids were emulsified and centrifuged at 3000 rpm. The supernatant was divided into aliquot, and stored at −70°C. The blood samples were treated with the procedure of 4°C 1 h, 37°C 1 min, and then centrifuged at 4°C 5000 rpm for 10 min. The serum was stored at –70°C.

### Cells and cell infection

Baby hamster kidney (BHK-21) cells were cultured in Dulbecco’s modified Eagle medium (DMEM; Invitrogen) supplemented with 10% fetal bovine serum (FBS; Hyclone). The well grown BHK-21 cells in 25 ml culture plate were inoculated with 1 ml OP fluids. Four milliliter sustaining medium was added per plate after 45 min adsorption. Cells were maintained at 37°C in humidified 5% CO_2_ environment for no more than 72 h. Cell status was observed every 3 h, and harvested when 95% CPE appeared. The infected cells should be harvested within 72 h. Cells without CPE would go on next cell infection cycle after three times of freeze-thaw [32]. Cell infection cycles were done no more than 10 times for each sample. Samples were identified as negative when no CPE was detected within 10 times of cell infection cycles.

### RNA extraction and RT-PCR detection

The treated OP fluids were used for RNA extraction with RNeasy Mini Kit (QIAGEN). The existence of viral RNA was measured by RT-PCR assay with the primers for *VP1*. Primers were designed based on the genome sequence of O/Akesu/58 with Oligo 7.0. The product length is 639 bp. The primers were as following:

Forward primer 5'-TAGTGCGGTTAAAGCTTTG-3',

Reverse primer 5'-GACATGTCCTCCTGCATCTG-3'.

The reverse transcription was performed in the volume of 20 μl containing 2 μg of total RNA, 0.5 mM dNTP each, 2.5 μM reverse primer, 4 μl 5 × AMV buffer, and 0.5U/μl AMV reverse transcriptase (TaKaRa).

The PCR was performed in the volume of 50 μl. The final concentration of each reagent was: 0.4 mM dNTP, 0.4 μM forward primer, 0.4 μM reverse primer, cDNA 6 μl, 10 × LA Buffer 5 μl, and LA Taq enzyme 0.05 U/μl (TaKaRa).

### T-A clone and sequencing of VP1

The RT-PCR products of *VP1* were purified with Gel DNA Extraction Kit (QIAGEN). T-A clone was performed with pGEM-T Easy vector (Promega). The ligase product was transformed into competent cells DH5α. The plasmids extracted with Plasmid Purification Kit (TaKaRa) were identified with PCR. The positive plasmid was sequenced by ABI-PRISM3730.

### Antibody detection

The total FMDV antibodies were detected with a commercial LPB-ELISA kit, and all procedures were performed according to the manufacturer’s instructions [33].

To detect the antibody level against 3ABC NSP of FMDV, a commercially available kit was used, and all procedures were carried out by following the manufacturer’s instructions [34].

### Sequence analysis

The nucleotide and protein sequences of the five isolate strains (GenBank ID: JQ693472, JQ693473, JQ693474, JQ693475, JQ693476) and O/Akesu/58 (GenBank ID: AJ131469) were aligned by MegAlign in DNAstar, and pairwise genetic distances were computed.

## Competing interests

The authors declare that they have no competing interests.

## Authors’ contributions

HC designed the study and performed the virus infection and the sampling work, and wrote the manuscript. YM advised on the study design, carried out the RT-PCR and sequencing work, assisted with the analysis of data and the manuscript. TL and GC were responsible for the preparation of animals and virus, and the sampling work. JD and JM carried out the detection of antibody levels and CPE detection. All authors read and approved the final manuscript.
